# The roles of risk perception, negative emotions and perceived efficacy in the association between COVID-19 infection cues and preventive behaviors: a moderated mediation model

**DOI:** 10.1186/s12889-022-14870-7

**Published:** 2023-01-16

**Authors:** Guangteng Meng, Qi Li, Xiaoyan Yuan, Ya Zheng, Kesong Hu, Bibing Dai, Xun Liu

**Affiliations:** 1 CAS Key Laboratory of Behavioral Science, Institute of Psychology, Chinese Academy of Sciences, Beijing, 100101 China; 2 Department of Psychology, University of Chinese Academy of Sciences, Beijing, 100049 China; 3 Beijing Key Laboratory of Learning and Cognition, School of Psychology, Capital Normal University, Beijing, 100048 China; 4 Big Data Group, Smart Platform R&D Department, Tianjin Meiteng Technology Co., Ltd, Tianjin, 300381 China; 5 Department of Psychology, Dalian Medical University, Dalian, 116044 China; 6 Department of Psychology, Lake Superior State University, Sault St. Marie, MI USA; 7 Department of Psychiatry and Psychology, School of Basic Medical Sciences, Tianjin Medical University, Tianjin, 300070 China

**Keywords:** COVID-19, Infection cues, Preventive behaviors, Risk perception, Negative emotions, Perceived efficacy

## Abstract

Preventive behaviors during the COVID-19 pandemic are especially critical to the protection of individuals whose family members or acquaintances have been infected. However, limited research has explored the influence of infection cues on preventive behaviors. This study proposed an interaction model of environment-cognitive/affective-behavior to elucidate the mechanism by which infection cues influence preventive behaviors and the roles of risk perception, negative emotions, and perceived efficacy in that influence. To explore the relationships among these factors, we conducted a cross-sectional online survey in 34 provinces in China during the first wave of the COVID-19 pandemic. A total of 26,511 participants responded to the survey, and 20,205 valid responses (76.2%) were obtained for further analysis. The moderated mediation results show that infection cues positively predicted preventive behaviors in a manner mediated by risk perception and negative emotions. Moreover, perceived efficacy moderated the influence of infection cues not only on preventive behaviors but also on risk perception and negative emotions. The higher the perceived efficacy, the stronger these influences were. These findings validated our model, which elucidates the mechanisms underlying the promoting effect of infection cues on preventive behaviors during the initial stage of the COVID-19 pandemic. The implications of these results for the COVID-19 pandemic and beyond are discussed.

## Introduction

The coronavirus disease 2019 (COVID-19) pandemic has disrupted the lives of everyone worldwide [1–3]. Local health authorities usually issue warning messages about infection cases, and such warning messages are regarded as one of the most used approaches to promoting the adoption of preventive behaviors during pandemics [4, 5]. Therefore, it is necessary to investigate whether and how infection cues promote the adoption of preventive behaviors during the COVID-19 pandemic.

To illustrate the mechanism by which warning messages influence behavioral reactions, the protective action decision model (PADM) was proposed to explain people’s actions in response to natural disasters [5], which has been applied in floods [6], hurricanes [7], and wildfires [8]. The PADM suggests that warning messages can elicit perceptual and emotional responses to threats, resulting in behavioral responses, and these processes depend on receiver characteristics, such as their beliefs. When people are confronted with urgent warnings, they estimate perceived risk and negative emotions regarding the threat and finally engage in adaptive behaviors [5]. However, warning messages during pandemics have received much less attention. Unlike natural disasters, pandemics can usually be persistent, in which infection cues play an important but obscure role. Therefore, a specialized model for infection cues in the context of the COVID-19 pandemic is still needed.

Based on the PADM, we herein propose the interaction model of environment-cognitive/affective-behavior to enhance the practical application of these theories in the specific context of the COVID-19 pandemic. This model elucidates the mechanism by which infection cues influence preventive behaviors. In this model, infection cues (I) were regarded as a kind of warning message that can directly make people aware of the infections of their relatives and friends. An individual’s risk perception of infection and negative emotions (P/E) is defined by their core cognitive and emotional reactions to the COVID-19 pandemic. Preventive behaviors related to the COVID-19 pandemic are considered behavioral reactions (A). Moreover, in this model, perceived efficacy, which is an important personal characteristic, plays a significant moderating role in the relationship between warning messages and an individual’s psychological reactions, such as alertness to infection cues and motivation to adopt protective behaviors.

### COVID-19 infection cues and preventive behaviors

Infection cues (I) are confirmed cases of COVID-19 among family members, friends, or acquaintances. Given that most disease transmission occurs among family members, friends, colleagues, and neighbors, which has been observed during various pandemics [9–11], the presence of infection cues indicates an increased likelihood of infection [12]. Preventive behaviors involve voluntary actions to avoid infection during an influenza pandemic [13], such as hygiene behaviors, mask wearing, social distancing, and uptake of vaccinations [14]. Empirical research has suggested that infection cues promote the adoption of preventive behaviors [15, 16]. Recently, people whose immediate family members, close friends, or relatives tested positive for COVID-19 were found to more frequently wear a facemask in public and clean the surfaces they touched [17]. Based on this evidence, we hypothesized that COVID-19-related infection cues serve as core warning messages and are positively associated with the adoption of preventive behaviors.

### Risk perception as a mediator

Risk perception involves how people subjectively assess the probability of a specific accident and how much they are concerned about the corresponding consequence [18]. People who receive infection cues are likely to perceive themselves as being at greater risk because they share both environmental and social surroundings with infected family members or acquaintances [16]. On the other hand, recent evidence suggests that risk perception could predict the adoption of preventive behaviors during the COVID-19 pandemic [19, 20]. Therefore, we hypothesized that risk perception could mediate the relationship between COVID-19-related infection cues and the adoption of preventive behaviors.

### Negative emotions as a mediator

Similarly, COVID-19-related infection cues may also promote the adoption of preventive behaviors by increasing the strength of emotional reactions, particularly those involving negative emotions. Converging evidence has shown that people who receive infection cues experience more negative emotions, such as anxiety, depression, and fear [21, 22]. Recently, increased anxiety and depression symptoms have also been found to co-occur with various behavioral changes, such as decreased physical activity [23]. In addition, fear induced by the pandemic can also motivate individuals to adopt protective measures, including social distancing and hand washing [24]. Hence, we hypothesized that negative emotions could also mediate the relationship between COVID-19-related infection cues and the adoption of preventive behaviors.

### Perceived efficacy as a moderator

In the interaction model of environment-cognitive/affective-behavior, perceived efficacy may play a moderating role in the relationship between infection cues and the adoption of preventive behaviors. Perceived efficacy is an individual’s belief in their ability to cope with specific risks [25, 26] and consists of self-efficacy and response efficacy [27]. First, perceived efficacy could influence how people process the warning messages they receive, which may trigger the perception that they are at greater risk [28]. People with low self-efficacy tend to adopt negative emotion-focused coping strategies to reduce their negative emotions rather than to solve problems [29]. A meta-analysis also confirmed that perceived efficacy could enhance the positive impact of risk appraisal on the adoption of preventive behaviors [30]. Therefore, while confronting infection cues, people who have higher levels of perceived efficacy may be more likely to engage in preventive behaviors.

### Current study

**Fig. 1:**
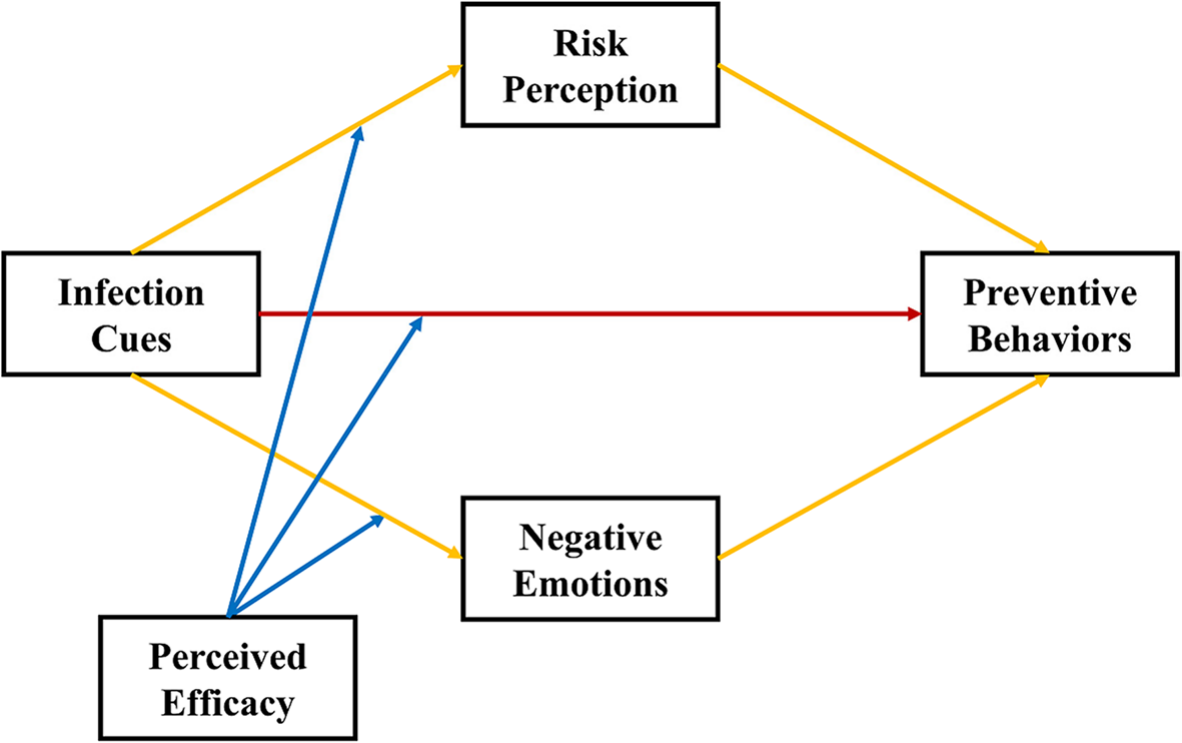
The hypothesized model. Note: H1 is in red, H2 is in yellow, and H3 is in blue

Building upon previous theories and findings, this study is among the first to investigate the mechanism underlying the association between infection cues and the adoption of preventive behaviors and the important roles of risk perception, negative emotions, and perceived efficacy in that relationship at the peak of the COVID-19 pandemic in China. The following major research hypotheses were addressed (Fig. 1): (H1) infection cues, such as having family members, friends, and acquaintances contract COVID-19, can promote stricter adherence to preventive behaviors; (H2) infection cues can increase people’s perceived level of risk and negative emotions and thereby increase their tendency to adopt preventive behaviors; and (H3) perceived efficacy can moderate the direct relationship between infection cues and the adoption of preventive behaviors and the moderating effects of risk perception and negative emotions. Specifically, the direct and moderated relationships between infection cues and preventive behaviors are stronger among individuals with high levels of perceived efficacy than among those with low levels of perceived efficacy.

## Methods

### Participants and procedures

The present study was approved by the Ethics Committee of the Institute of Psychology of the Chinese Academy of Sciences. Data collection was conducted from February 4 to 6, 2020. During this period, the total number of confirmed cases of COVID-19 exceeded 20,000 in China. A national cross-sectional web-based survey was conducted involving a nonprobability (convenience) sample of the Chinese population with the Tencent online platform. We provided a quick response code that participants could use to access the electronic version of the survey, which they could then complete, submit, and share. Therefore, the data were collected by snowball sampling through repeated one-to-many sharing on social media. After the participants and/or their legally authorized representatives read and signed the informed consent, we asked them to respond to 11 items regarding infection cues, risk perception, negative emotions, perceived efficacy, and preventive behaviors. In the present study, these items were chosen to reflect these variables in the context of the COVID-19 pandemic, and most of them had good or acceptable reliability. To ensure the quality of the data from the respondents, we excluded 6306 of the 26,511 surveys based on two criteria. First, 4765 surveys from individuals who completed the full survey in less than 1 min were excluded because answering too quickly may be the result of failing to read the questions carefully. Second, questions for which the participants were required to choose a certain option were also included. In total, 1,541 surveys were excluded due to incorrect answers to these questions, which indicated that those respondents did not read the questionnaire items carefully. After these exclusions, 20,205 (76.21%) surveys remained for inclusion in the present analyses. Table 1 summarizes the sample.

**Table 1:** Demographic characteristics of the sample (*N* = 20,205), n (%)

Characteristic	With infection cues (*n* = 5527)	Without infection cues (*n* = 14,678)	Total (*N* = 20,205)
**Age (years)**			
12–17	1593 (28.8)	2161 (14.7)	3754 (18.6)
18–25	1868 (33.8)	5283 (36.0)	7151 (35.4)
26–35	1483 (26.8)	4545 (30.9)	6028 (29.8)
36–45	415 (7.5)	1967 (13.4)	2382 (11.8)
46–61	168 (3.0)	722 (4.9)	890 (4.4)
**Gender**			
Female	1722 (31.2)	6198 (42.2)	7920 (39.2)
Male	3805 (68.8)	8480 (57.8)	12,285 (60.8)
**Education**			
High school or lower	2114 (38.2)	6247 (42.6)	8361 (41.4)
College/technical school	1414 (25.6)	3246 (22.1)	4660 (23.1)
University undergraduate degree	1732 (31.3)	4643 (31.6)	6375 (31.6)
Master’s degree or higher	267 (4.8)	542 (3.7)	809 (4.0)
**Occupation**			
Student	1524 (27.6)	4994 (34.0)	6518 (32.3)
Enterprise employee	1802 (32.6)	4331 (29.5)	6133 (30.4)
Self-employed	530 (9.6)	1735 (11.8)	2265 (11.2)
Factory/agricultural worker	484 (8.8)	1625 (11.1)	2109 (10.4)
Civil servant	620 (11.2)	688 (4.7)	1308 (6.5)
Professional	341 (6.2)	676 (4.6)	1017 (5.0)
Others	226 (4.1)	629 (4.3)	855 (4.2)
**Region of China**			
East China (e.g., Shandong)	1176 (21.3)	4113 (28.0)	5285 (26.2)
North China (e.g., Beijing)	1750 (31.7)	3963 (27.0)	5713 (28.3)
Central China (e.g., Hubei)	529 (9.6)	1608 (11.0)	2137 (10.6)
South China (e.g., Guangdong)	305 (5.5)	1907 (13.0)	2212 (10.9)
Northeast China (e.g., Liaoning)	1574 (28.5)	2070 (14.1)	3644 (18.0)
Northwest China (e.g., Xinjiang)	80 (1.4)	402 (2.7)	482 (2.4)
Southwest China (e.g., Chongqing)	113 (2.0)	615 (4.2)	728 (3.6)

## Measures

### COVID-19 infection cues

COVID-19 infection cues were assessed with the following question, to which the respondents were asked to provide a yes or no answer: “Has someone among your family members, friends, and acquaintances been diagnosed with COVID-19 by a local hospital or the health department?”.

### Risk perception

Risk perception was measured with two questions: “In your opinion, how contagious is COVID-19?” and “In your opinion, how likely are you to contract COVID-19?” For these two items, the respondents were asked to evaluate the degree of risk they perceived to be associated with COVID-19, ranging from 1 = very low to 7 = very high. The overall risk perception score was determined by summing the scores for these two questions, with higher scores indicating greater risk perception. The Cronbach’s α was 0.59 in the present study.

### Negative emotions

Existing research recognizes the critical role played by anxiety, depression, and fear in negative emotions [18, 31]. We have referred to the Positive and Negative Affect Schedule (PANAS) and asked people to rate the intensity of three negative emotion words and give each word equal weight to calculate the total score. In addition, previous studies usually measure the related symptoms during the last one (e.g., Self-Rating Anxiety Scale) or two weeks (e.g., Patient Health Questionnaire-9). Considering the circumstances, we asked people to report their experience across the last ten days, which falls in between. Negative emotions were assessed with three questions: “In the last 10 days, what intensity of anxiety have you experienced?”, “In the last 10 days, what intensity of depression have you experienced?”, and “In the last 10 days, what intensity of fear have you experienced?” For these three items, the respondents were asked to rate the intensity of their negative emotions from 1 = very low to 7 = very high. The total score for negative emotions was the sum of the scores for the three questions. Higher scores indicated a higher level of negative emotions. The Cronbach’s α was 0.89 in the present study.

### Perceived efficacy

Perceived efficacy, which consists of response efficacy and self-efficacy, was assessed with four items taken from a well-established perceived efficacy scale [32]: “I believe the pandemic will be fully controlled in the foreseeable future”; “I am confident that the pandemic will be overcome”; “To cope with the pandemic, I can discriminate between true information and rumours about COVID-19”; and “To combat the pandemic, I do not post or forwards any messages about COVID-19 that have not been officially confirmed.” These items assess people’s beliefs in both preventive behaviors and their own ability to carry them out. For these four items, the respondents were asked to rate their perceived efficacy from 1 = strongly disagree to 7 = strongly agree. The total score was equal to the sum of the scores for these four items, and higher scores indicated a higher level of perceived efficacy. The Cronbach’s α was 0.79 in the present study.

### Preventive behaviors

Preventive behaviors were assessed with one item: “I have adopted or will adopt COVID-19 preventive behaviors (e.g., wearing masks, washing hands, keeping social distancing, taking vaccinations, and so on) as soon as they are available.” The respondents were asked to evaluate the extent to which they would adopt these preventive behaviors from 1 = strongly disagree to 7 = strongly agree.

### Statistical analyses

Statistical analyses were conducted using SPSS, version 26.0 (IBM Corp). Descriptive analyses were carried out using the mean (SD) for quantitative variables and frequency (%) for qualitative variables. In this study, we first conducted bivariate correlation analyses of these variables to examine the general relationships among infection cues, risk perception, negative emotions, perceived efficacy, and preventive behaviors. Then, we used the PROCESS macro (Model 8) to test our moderated mediation model, as suggested by Hayes [33]. The PROCESS macro for SPSS is an observed variable ordinary least squares and logistic regression path analysis modeling tool that can provide estimates of model coefficients and assessments of the direct and/or indirect effects of variables in the model. In addition, the PROCESS models also use a bootstrapping procedure (a total of 5000 resamples in the present study) to generate a robust standard error for the parameter estimation and the bias-corrected 95% CIs associated with the significance of indirect effects, regardless of the normality of the sample distribution. Specifically, PROCESS Model 8 included three models [34], in which risk perception, negative emotions, and preventive behaviors were the dependent variables. This model addressed the effect of the interaction between infection cues and perceived efficacy on risk perception (the first aspect of mediation), the effect of the interaction between infection cues and perceived efficacy on negative emotions (the second aspect of mediation), and the effect of the interaction between infection cues and perceived efficacy on the adoption of preventive behaviors (the residual direct relationship). Interaction effects and conditional indirect and direct effects can be identified when the confidence intervals do not contain zero. In these analyses, we controlled for relevant sociodemographic covariates (i.e., gender, age, and education) by entering them as predictor variables into regression equations. Thus, these covariates were not underlying factors explaining the direct and indirect associations of infection cues with the adoption of preventive behaviors.

## Results

### Preliminary analyses

The descriptive statistics and correlation matrices are presented in Table 2. Infection cues, preventive behaviors, risk perception, and negative emotions were positively correlated with each other. Perceived efficacy was positively correlated with preventive behaviors and risk perception and negatively correlated with infection cues and negative emotions.

**Table 2:** Descriptive statistics and correlations among variables (*N* = 20,205)

Variables	Mean	SD	1	2	3	4	5	6	7	8
1. Gender	-	-	1							
2. Age	26.09	9.67	-0.231***	1						
3. Education	-	-	-0.047***	0.159***	1					
4. Infection cues	-	-	0.101***	-0.151***	0.030***	1				
5. Preventive behaviors	5.08	1.73	-0.017*	0.060***	0.049***	0.047***	1			
6. Risk perception	7.67	3.23	-0.012	-0.023**	0.046***	0.269***	0.205***	1		
7. Negative emotions	11.48	5.32	-0.026***	0.016*	0.053***	0.264***	0.186***	0.459***	1	
8. Perceived efficacy	22.88	4.57	-0.073***	0.162***	0.047***	-0.136***	0.396***	0.042***	-0.015*	1

Gender: 0 = female, 1 = male. Education: 1 = High school or lower, 2 = College/technical school, 3 = University undergraduate degree, 4 = Master’s degree or higher. Infection cues: 0 = no, 1 = yes. *P < 0.05, **P < 0.01, ***P < 0.001

### Moderated mediation

**Fig. 2:**
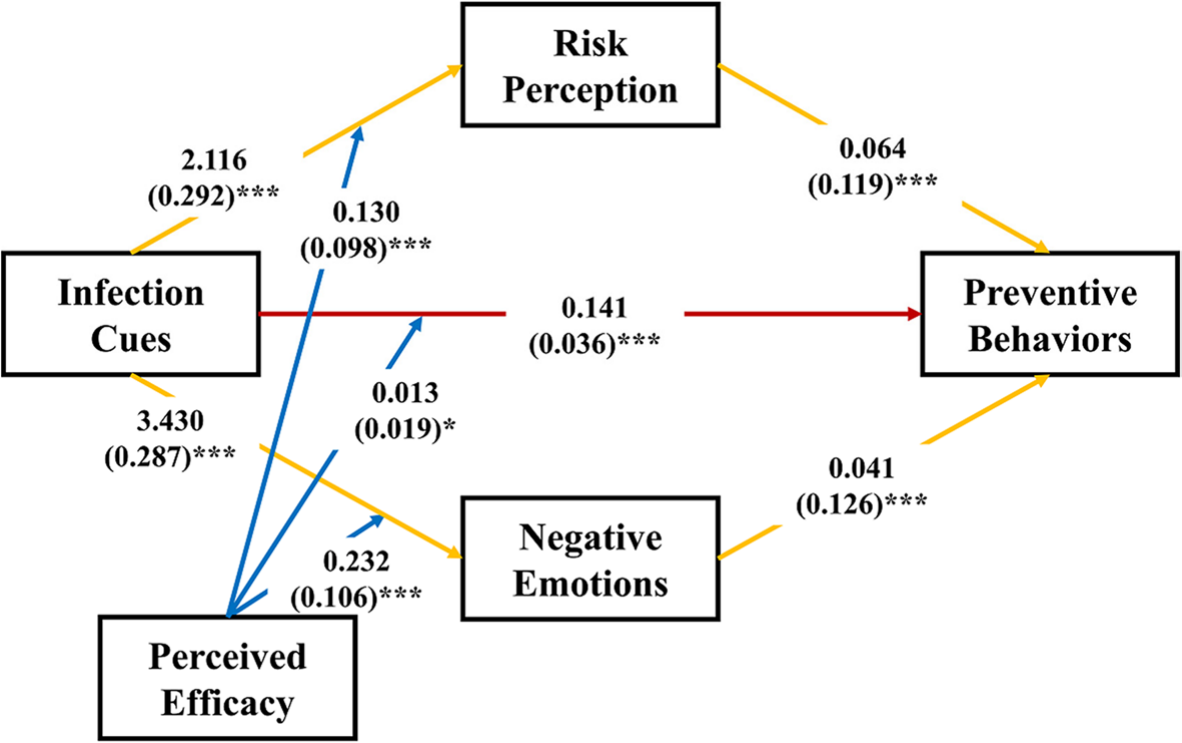
The moderated mediation model. Note: The values shown are the unstandardized (standardized) coefficients. *P < .05. **P < .01. ***P < .001

The main results generated by the SPSS PROCESS macro are presented in Table 3; the results consisted of five parts: Model 1, Model 2, Model 3, the conditional direct effect analysis, and the conditional indirect effect analysis. Model 1 tested the effects of infection cues and perceived efficacy on risk perception. Model 2 examined the effects of infection cues and perceived efficacy on negative emotions. Model 3 investigated the effects of infection cues, risk perception, negative emotions, and perceived efficacy on preventive behaviors. Model 1 (*F*_6,20198_ = 325.277, R^2^ = 0.088, *P* < 0.001), Model 2 (*F*_6,20198_ = 906.005, R^2^ = 0.239, *P* < 0.001), and Model 3 (*F*_8,20196_ = 662.574, R^2^ = 0.208, *P* < 0.001) showed that infection cues positively predicted the adoption of preventive behaviors (B = 0.141, β = 0.036, *P* < 0.001) after controlling for gender, age, and education, which supported H1 (Fig. 2). In addition, infection cues positively predicted risk perception (B = 2.116, β = 0.292, *P* < 0.001), and risk perception positively predicted the adoption of preventive behaviors (B = 0.064, β = 0.119, *P* < 0.001). Infection cues positively predicted negative emotions (B = 3.430, β = 0.287, *P* < 0.001), and negative emotions positively predicted the adoption of preventive behaviors (B = 0.041, β = 0.126, *P* < 0.001), supporting H2.

**Table 3:** Conditional process analysis of the proposed moderated mediation model

	β	B (SE)	*t*	95% CI lower	95% CI upper
**Model 1** Outcome: Risk perception					
Gender	-.035***	-0.233 (0.046)	-5.099	-0.323	-0.144
Age	-.007	-0.002 (0.002)	-0.983	-0.007	0.002
Education	.036***	0.108 (0.020)	5.332	0.068	0.147
Infection cues	.292***	2.116 (0.050)	42.167	2.018	2.214
Perceived efficacy	.026***	0.054 (0.005)	11.133	0.044	0.063
Infection cues × Perceived efficacy	.098***	0.130 (0.011)	12.155	0.109	0.151
**Conditional indirect effect 1**					
M – 1 SD	.023	0.097 (0.007)		0.084	0.112
M	.035	0.135 (0.009)		0.118	0.153
M + 1 SD	.046	0.173 (0.012)		0.150	0.197
**Model 2** Outcome: Negative emotions					
Gender	-.043***	-0.472 (0.076)	-6.238	-0.520	-0.324
Age	.040***	0.022 (0.004)	5.572	0.014	0.030
Education	.036**	0.176 (0.033)	5.289	0.111	0.242
Infection cues	.287***	3.430 (0.083)	41.340	3.267	3.593
Perceived efficacy	-.044	0.012 (0.008)	1.490	-0.004	0.028
Infection cues × Perceived efficacy	.106***	0.232 (0.018)	13.100	0.197	0.266
**Conditional indirect effect 2**					
M – 1 SD	.023	0.097 (0.007)		0.083	0.111
M	.036	0.140 (0.009)		0.122	0.159
M + 1 SD	.050	0.183 (0.013)		0.160	0.209
**Model 3** Outcome: Preventive behaviors					
Gender	.014**	0.051 (0.023)	2.217	0.006	0.096
Age	.002	0.000 (0.001)	0.365	-0.002	0.003
Education	.018**	0.029 (0.010)	2.900	0.010	0.049
Infection cues	.036***	0.141 (0.027)	5.317	0.089	0.193
Risk perception	.119***	0.064 (0.004)	16.585	0.056	0.072
Negative emotions	.126***	0.041 (0.002)	17.514	0.036	0.045
Perceived efficacy	.387***	0.150 (0.002)	61.801	0.145	0.155
Infection cues × Perceived efficacy	.019*	0.013 (0.005)	2.464	0.003	0.024
**Conditional direct effect**					
M – 1 SD	.017	0.081 (0.033)		0.016	0.146
M	.036	0.141 (0.027)		0.089	0.193
M + 1 SD	.055	0.202 (0.039)		0.125	0.278

Results obtained with bootstrapping (*n* = 5000). Conditional indirect effect 1 was infection cues → risk perception → preventive behaviors. Conditional indirect effect 2 was infection cues → negative emotions → preventive behaviors. *β* Standardized Coefficients, *B* Unstandardized Coefficients, *SE* Standard Error, *CI* Confidence Interval. *P < 0.05, **P < 0.01, ***P < 0.001

The interaction between infection cues and perceived efficacy had a significant effect on the adoption of preventive behaviors (B = 0.013, β = 0.019, *P* = 0.01). Meanwhile, the interaction between infection cues and perceived efficacy had significant effects on risk perception (B = 0.130, β = 0.098, *P* < 0.001) and negative emotions (B = 0.232, β = 0.036, *P* < 0.001), supporting H3. Furthermore, as shown by the results regarding the conditional direct effect (Table 3), all three of the conditional direct effects (based on the moderator values at the mean plus and minus one standard deviation) were positive and significantly different from zero, indicating that infection cues had a stronger direct predictive role in individuals with high levels of perceived efficacy than in individuals with low levels of perceived efficacy. As shown in the result for conditional indirect effect 1, all three of the conditional indirect effects were positive and significantly different from zero. This finding suggested that the indirect effect of infection cues on preventive behaviors through risk perception was higher among individuals with a high level of perceived efficacy than among individuals with a low level of perceived efficacy due to the interaction of infection cues and perceived efficacy. Meanwhile, as shown by the result for conditional indirect effect 2, these three conditional indirect effects were positive and significantly different from zero, which indicated that the indirect influence of infection cues on preventive behavior through negative emotions was stronger in the individuals with high levels of perceived efficacy than in those with low levels of perceived efficacy.

## Discussion

### Principal results

In this study, we found that individuals who received COVID-19-related infection cues exhibited more preventive behaviors. Such cues could also increase both their risk perception and negative emotions, indirectly enhancing their adoption of preventive behaviors. Furthermore, perceived efficacy generally strengthened the effects of other factors, including infection cues, risk perception, and negative emotions, promoting the adoption of preventive behaviors. These findings validate the interaction model of environment-cognitive/affective-behavior, in which the psychological mechanism involves the promotion of preventive behaviors by infection cues in the context of the COVID-19 pandemic.

### Impact of infection cues on the adoption of preventive behaviors

Our results indicate that people who receive infection cues are more inclined to engage in preventive behaviors. This finding is in line with several previous studies [17, 35]. Once a family member, friend, or acquaintance tests positive for COVID-19, people imminently feel the risk of the COVID-19 pandemic, and thus they engage in more preventive behaviors. In contrast, people who have not received infection cues have vague perceptions and ambiguous attitudes regarding the COVID-19 pandemic because of the limited warning messages primarily based on hearsay. In summary, infection cues are an important starting point from which people recognize, reappraise, and react to a pandemic.

### Mediating effects of risk perception and negative emotions

As hypothesized, we found that infection cues facilitated preventive behaviors via increased levels of both risk perception and negative emotions. Part of this finding is in line with that of a previous study that suggested that warning messages obtained through social media can increase the adoption of preventive behaviors via increased risk perception and negative emotions [36]. On the one hand, driven by the desire for self-protection, people with higher levels of risk perception are more likely to take comprehensive precautionary measures against infection [37]. On the other hand, infection cues result in a higher level of negative emotions because people worry about the health and safety of their family members, friends, and acquaintances [38]. Therefore, a stable moderate level of negative emotions during the pandemic is beneficial because it reminds individuals to pay more attention to the pandemic, seek effective preparatory measures, and engage in preventive behaviors.

### Perceived efficacy moderating the effect of infection cues on preventive behaviors

Another important finding in the current study pertains to the moderators. First, for individuals with high levels of perceived efficacy, infection cues better promote the adoption of preventive behaviors. People with higher levels of perceived efficacy favorably estimate the effectiveness of preventive behaviors [39] and hence actively take them as soon as infection cues come up. Second, the positive effect of infection cues on risk perception is significantly enhanced in individuals with high levels of perceived efficacy. People with high levels of perceived efficacy pay more attention to the fact that infection cues have occurred in their social surroundings. Third, the effect of infection cues on negative emotions was enhanced in individuals with high levels of perceived efficacy. It has been suggested that people with low levels of perceived efficacy believe that they are unable to change the level of threat and cope with their negative emotions through maladaptive responses, such as denial [29]. We suppose that it is adaptive for people to have a moderate level of negative emotions to be alarmed.

### Interaction model of environmental-cognitive/affective-behavior

The resulting moderated mediation model presented here facilitates a better understanding of the relationship between infection cues and the adoption of preventive behaviors. According to construal level theory [40], individuals who receive concrete messages retain more information than those who receive abstract messages, and their stronger perceptions and emotional reactions lead to more changes in their behavior. Instead of abstract information such as TV news and broadcasts, infection cues provide a clear signal with detailed and specific warning messages that a particular person in people’s surroundings contracted COVID-19. The subsequent vivid details of the symptoms can elicit a more firmly rooted belief in the existence of the pandemic. In such a context of uncertainty, individuals feel more negative emotions and take the necessary actions to protect both themselves and their families. In addition, individuals who believe that they can take action to avoid infection will exhibit stronger psychological and behavioral reactions to infection cues.

### Implications

In the context of a novel pandemic, effective communication of warning messages can help people adopt preventive behaviors. However, such communication remains challenging for governments and health authorities. The findings of the current study have several clinical and public health implications. First, infection cues could draw attention to the need to implement preventive behaviors, but this information is usually delivered through natural social networks with uncertainty and error probability. We suggest that governments could help people efficiently check and update the status of infection cues by providing information about the confirmed cases to their families, communities, and workplaces routinely. Second, a reasonable level of risk perception and a moderate level of negative emotions about COVID-19 are beneficial for both governments and individuals until full control over the pandemic has been achieved. People’s excessive optimism and consequent underestimation of the level of risk may lead to a failure to control the pandemic. Unlike natural disasters such as earthquakes and hurricanes, pandemics are persistent and involve a contagious disease. Appropriate levels of risk perception and negative emotions are conducive to controlling the COVID-19 pandemic. Third, positive guidance should be provided to enhance individuals' confidence in preventive behaviors. Otherwise, individuals might lose their sense of control and turn to stigmatization and aggression.

### Limitations

Several limitations of the present study must be noted. First, the current study adopted the simple moderated mediation model rather than structural equation modeling, since there were less than three measurement indicators for most variables. Future studies can appropriately increase the number of measurement indicators for each variable. Longitudinal design and structural equation model analysis methods could be adopted to explore the interaction of risk perception, negative emotions, perceived effects, infection cues, and preventive behaviors over time, which would be a very interesting and challenging topic. Second, the current study only employed a single question of infection cues on family members, friends, and acquaintances. However, infection cues covered the COVID-19 status of the neighborhood at various levels, such as district, city, and region, for residence and work. In future studies, it is necessary to design infection cues from a multidimensional perspective to investigate their influence on preventive behavior more comprehensively. Third, this study focused on several voluntary preventive behaviors. We suggest that future work should take mandatory preventive measures (e.g., home isolation and lockdown) and alternative social contact (e.g., virtual meetings) into consideration and examine their potential impacts. In addition, both information systems and health campaigns regarding the pandemic situation can influence how people identify infection cues and consequently affect preventive behaviors, risk perception, negative emotions, and perceived efficacy. We suggest that future studies and policymakers pay more attention to the impacts of information systems and health campaigns.

## Conclusions

This study collected large-scale data during the COVID-19 pandemic in China and determined the mechanism by which infection cues promote the adoption of preventive behaviors. Our findings demonstrate that individuals who receive infection cues engage in more preventive behaviors due to their increased risk perception and negative emotions and that high levels of perceived efficacy further enhance these effects. This study identified the mechanism by which infection cues contributed to the adoption of preventive behaviors during the COVID-19 pandemic and suggests that an early warning and support system based on the dynamic surveillance of infection cues should be established.

## Data Availability

The datasets generated and analysed during the current study are available in the OSF repository, [https://osf.io/g9stp/].
